# Inefficient co-feeding transmission of *Borrelia afzelii* in two common European songbirds

**DOI:** 10.1038/srep39596

**Published:** 2017-01-05

**Authors:** Dieter J. A. Heylen, Hein Sprong, Aleksandra Krawczyk, Natalie Van Houtte, Dolores Genné, Andrea Gomez-Chamorro, Kees van Oers, Maarten J. Voordouw

**Affiliations:** 1 Evolutionary Ecology Group, Department of Biology, University of Antwerp, Belgium; 2 Laboratory for Zoonoses and Environmental Microbiology, National Institute for Public Health and Environment (RIVM), Bilthoven, The Netherlands; 3 Laboratory of Ecology and Evolution of Parasites, Institute of Biology, University of Neuchâtel, Switzerland; 4 Department of Animal Ecology, Netherlands Institute of Ecology (NIOO-KNAW), Wageningen, The Netherlands

## Abstract

The spirochete bacterium *Borrelia afzelii* is the most common cause of Lyme borreliosis in Europe. This tick-borne pathogen can establish systemic infections in rodents but not in birds. However, several field studies have recovered larval *Ixodes ricinus* ticks infected with *B. afzelii* from songbirds suggesting successful transmission of *B. afzelii*. We reviewed the literature to determine which songbird species were the most frequent carriers of *B. afzelii*-infected *I. ricinus* larvae and nymphs. We tested experimentally whether *B. afzelii* is capable of co-feeding transmission on two common European bird species, the blackbird (*Turdus merula*) and the great tit (*Parus major*). For each bird species, four naïve individuals were infested with *B. afzelii*-infected *I. ricinus* nymphal ticks and pathogen-free larval ticks. None of the co-feeding larvae tested positive for *B. afzelii* in blackbirds, but a low percentage of infected larvae (3.33%) was observed in great tits. Transstadial transmission of *B. afzelii* DNA from the engorged nymphs to the adult ticks was observed in both bird species. However, BSK culture found that these spirochetes were not viable. Our study suggests that co-feeding transmission of *B. afzelii* is not efficient in these two songbird species.

The tick-borne spirochete bacterium *Borrelia afzelii* is the most common etiological agent of Lyme borreliosis (LB) in Europe^1^,^2^,^3^. This pathogen is transmitted by *Ixodes ricinus* ticks and is adapted to infect rodent reservoir hosts^3^,^4^,^5^,^6^,^7^. In these hosts, *B. afzelii* establishes a long-term, systemic infection that facilitates high rates of host-to-tick transmission^6^,^8^,^9^,^10^,^11^. In contrast to bird-adapted *Borrelia* species such as *B. garinii* and *B. valaisiana*, experimental infection studies with blackbirds, pheasants, and great tits have shown that *B. afzelii* is not able to establish a systemic infection in these bird species^12^,^13^,^14^. The ability of *B. afzelii* to infect rodent but not avian hosts (and vice versa for the bird-adapted *Borrelia* species) appears to be mediated by the vertebrate complement system^15^,^16^. Thus, the general consensus is that *B. afzelii* is unable to use avian hosts to infect new ticks^1^,^2^,^3^,^17^.

Recent field studies on birds have questioned this consensus of whether *B. afzelii* is strictly incompatible with avian hosts. Many species of birds are frequently exposed to *B. afzelii*-infected *I. ricinus* nymphs^18^,^19^,^20^,^21^,^22^,^23^,^24^. More importantly, *B. afzelii*-infected larval ticks have been recovered from a number of bird species including *Fringilla coelebs* L., *Troglodytes troglodytes* L., *Parus major* L., *Turdus merula* L., and *Turdus iliacus* L. (see Table 1). Given that vertical transmission of LB pathogens is thought to be rare in *Ixodes* ticks^25^,^26^,^27^, these observations suggest that these larval ticks acquired *B. afzelii* spirochetes from avian hosts.

Co-feeding transmission is one strategy by which *B. afzelii* might infect larval ticks feeding on avian hosts. This mode of transmission occurs when infected and uninfected ticks feed in close spatial and temporal proximity on the same host^28^,^29^,^30^. A number of studies have documented co-feeding transmission of *B. afzelii* on competent rodent reservoir hosts^28^,^31^,^32^,^33^,^34^. The observation that this mode of transmission can occur in the absence of a systemic infection raised the hypothesis that co-feeding transmission could allow *Borrelia* pathogens to evade the hostile immune system of otherwise incompetent hosts^29^,^30^,^35^. For example, co-feeding transmission of *B. afzelii* and *B. garinii* has been documented on ungulates, which are believed to be refractory to systemic infection^36^,^37^. An experimental infection study using a Japanese strain of *B. garinii* demonstrated co-feeding transmission on laboratory mice^38^. However, an alternative explanation for this study is that this strain actually belonged to the closely related but rodent-adapted *B. bavariensis*, as this species was recently shown to be widespread in Asia, including Japan^39^.

The purpose of the present study was to test whether *B. afzelii* can use co-feeding transmission to infect *I. ricinus* larval ticks on two different species of songbird: the blackbird (*Turdus merula*) and the great tit (*Parus major*). We chose these two songbird species because they are common in Europe, are often exposed to immature *I. ricinus* ticks in nature, and they are highly competent reservoir hosts for bird-adapted *Borrelia* genospecies. The blackbird can amplify *B. garinii, B. valaisiana* and *B. turdi*^24^,^40^,^41^ and the great tit can amplify *B. garinii*^12^. In addition, we performed a literature review to determine how often songbirds carry *B. afzelii*-infected immature *I. ricinus* ticks in nature.

## Results

### Blackbird experiment

In the blackbird experiment, each of the four birds was infested with 11–12 nymphs before being infested with 40–50 co-feeding larvae 24 hours later. The challenge nymphs had been randomly selected from a population where the percentage of infected nymphs was 68.1% (47 infected/69 total). For the blackbirds, the nymphal and larval attachment rates (mean ± standard deviation) were 93.7 ± 12.5% per bird and 96.5 ± 4.7% per bird, respectively. A total of 20 engorged challenge nymphs and 128 engorged co-feeding larvae were recovered (mean ± standard deviation: 5.0 ± 0.8 nymphs per bird and 32 ± 12 larvae per bird). The engorged challenge nymphs were allowed to moult into adult ticks, which were tested using qPCR to determine whether the birds had been exposed to *B. afzelii*. A total of 17 challenge nymphs and 90 co-feeding larvae were tested for the four blackbirds (Table 2).

Two of the four blackbirds produced 2 and 4 infected adult ticks (Table 2) indicating that they were properly challenged. The presence of *B. afzelii* in 6 adult ticks suggests that there was nymph-to-adult transtadial transmission but we do not know whether these spirochetes were dead or alive. The other two birds produced 2 and 4 uninfected adult ticks (Table 2). Given that the estimated proportion of infected challenge nymphs was 0.681, the probability that these two birds would produce 6 uninfected adult ticks is (1–0.681)^6^ = 0.001. Our method of estimating nymphal attachment suggests that 9 and 11 challenge nymphs attached to these two birds. The probability that these two birds were infested with at least one *B. afzelii*-infected nymph is therefore very high (0.9999659 and 0.9999965, respectively). Thus we are confident that all four birds encountered at least one *B. afzelii*-infected nymph. However, none of the 90 xenodiagnostic larval ticks (tested as engorged larvae or as flat nymphs) that had co-fed with the challenge nymphs tested positive for *B. afzelii* (Table 2).

All ticks that had fed on the blackbirds and that had tested positive for *B. afzelii* on the qPCR were sequenced with respect to the *ospC* gene and the 5S-23S (rrfA-rrlB) intergenic spacer (IGS) region gene. We obtained 3 *ospC* sequences and 5 IGS sequences and all of them belonged to *B. afzelii*. This sequencing work confirms that the nymphs used to challenge the blackbirds were infected with *B. afzelii*.

### Great tit experiment

In the great tit experiment, each of the four birds was infested with 11–12 nymphs before being infested with 40–50 co-feeding larvae 24 hours later. The challenge nymphs had been randomly selected from a population where the percentage of infected nymphs was 91.5% (130 infected/142 total). For the great tits, the nymphal and larval attachment rates (mean ± standard deviation) were 58.3 ± 21.5% per bird and 85.6 ± 9.8% per bird, respectively. A total of 16 engorged challenge nymphs and 115 engorged co-feeding larvae were recovered (mean ± standard deviation: 4.0 ± 2.7 nymphs per bird and 28.8 ± 6.8 larvae per bird). The engorged challenge nymphs were either tested directly or were allowed to moult into adult ticks. A total of 16 challenge nymphs and 90 co-feeding larvae were tested for the four great tits (Table 2).

Analysis of the challenge ticks showed that all four great tits had been exposed to *B. afzelii* (2, 3, 8, and 2 infected ticks per bird; Table 2). Three of the 76 xenodiagnostic larval ticks (tested as engorged larvae) that had co-fed with the challenge nymphs tested positive for *B. afzelii*, but the pathogen was not detected in any of the 14 nymphs (moulted from the engorged larvae) (Table 2). Four of the five adult ticks obtained from three birds tested positive for *B. afzelii* based on the qPCR (Table 2), but the culture of these ticks in BSK-II medium did not yield any viable spirochetes.

### Summary of the infection experiments

Overall, the *B. afzelii*-infection rates in co-feeding larvae were low in both blackbirds (0.00% = 0/90) and great tits (3.33% = 3/90). In summary, we found limited co-feeding transmission of *B. afzelii* for the two bird species used in this study. We emphasize that our sample size was limited with only 4 individuals for each bird species.

### Literature review

Our review of the literature found 13 of 19 studies in which *B. afzelii* has been reported in songbird-derived *I. ricinus* ticks. Seven species of songbird could play a role in the transmission of *B. afzelii* to larval *I. ricinus* ticks (Table 1). The hosts that were most often reported to have *B. afzelii*-infected larvae were the European robin (*Erithacus rubecula*) and the great tit (2 studies). When considering birds that carried *B. afzelii*-infected nymphal ticks, we found 20 different bird species, of which the blackbird (7 studies), songthrush (*Turdus philomenos*) (6 studies), dunnock (*Prunella modularis*) (5 studies), European robin (5 studies), and great tit (4 studies) were most often reported.

## Discussion

Our study suggests that the rodent-adapted Lyme disease pathogen, *B. afzelii*, cannot use co-feeding transmission as an efficient strategy to infect naive ticks on two species of songbird. There was no co-feeding transmission of *B. afzelii* on the four blackbirds and only three larval ticks acquired *B. afzelii* via co-feeding transmission on the four great tits. The efficiency of co-feeding transmission of *B. afzelii* on the great tit was therefore low (3/90 = 3.33%). In contrast, the isolate of *B. afzelii* used in the great tit experiment (isolate NE4049; also referred to as *ospC* strain A10) has high co-feeding transmission (> 50.00%) on competent rodent reservoir hosts, and in these hosts there is successful trans-stadial transmission^32^,^34^. We acknowledge that one limitation of the current study is the small sample size (n = 8 birds). However, we point out that studies with similar sample sizes have detected co-feeding transmission of *B. afzelii* on rodents^28^,^32^,^33^. Recent theoretical studies have shown that co-feeding transmission makes a modest contribution to the reproductive number (R_0_) of *B. burgdorferi* pathogens^42^,^43^,^44^. Specifically, a co-feeding transmission efficiency of 50.0% increases the R_0_ value by 2.07–6.68% depending on a variety of ecological factors^42^. These analyses suggest that a co-feeding transmission efficiency of 3.33% would have a negligible effect on the R_0_ of *B. afzelii*. In summary, *B. afzelii* is transmitted efficiently via co-feeding transmission on rodent hosts but not on the two bird species investigated. Studies on *B. afzelii* in laboratory rodents have shown that strains differ in the efficacy of co-feeding transmission^32^,^34^. Studies on *B. burgdorferi* in North American passerines have shown that reservoir competence can vary widely between bird species^45^,^46^,^47^. We therefore emphasize that we cannot generalize these results to other strains of *B. afzelii* and other songbird species.

Our study also found evidence that avian blood is borreliacidal for *B. afzelii*. For the blackbirds, the probability that two birds would produce six uninfected adult ticks was highly unlikely (p = 0.001), given that an independent sample suggested that 68.1% (47 infected/69 total) of the challenge nymphs were infected with *B. afzelii* before feeding on these birds. Our results are similar to a previous study where *B. afzelii* was cleared from *I. ricinus* challenge nymphs after they had fed on pheasants, whereas bird-adapted *Borrelia* species were not cleared from the challenge nymphs^12^. Additional evidence for the borreliacidal effects of avian blood on *B. afzelii* was our demonstration using BSK-II cultures that none of the qPCR-positive adult ticks that had fed as nymphal ticks on the great tits contained viable spirochetes. Previous work has shown that the ability to detect *Borrelia* infections by culturing ticks in BSK media is similar to PCR-based methods^48^. This result suggests that our qPCR assay is detecting dead spirochetes in the adult ticks and shows the limitations of using DNA-based methods to infer the reservoir competence of a particular host species. Further studies using other combinations of pathogen strains and songbird species should investigate the generality of whether avian blood kills *B. afzelii* in *I. ricinus* during tick blood feeding.

Numerous field studies have shown the association of *B. afzelii* with rodent reservoir hosts^4^,^5^,^6^,^49^,^50^ and of *B. garinii* and *B. valaisiana* with avian reservoir hosts^7^,^12^,^13^,^21^,^24^,^40^,^41^,^51^,^52^,^53^. The cycling of *B. afzelii* and *B. garinii* in different classes of vertebrate hosts is also supported by studies on wild *I. ricinus* nymphs, which have shown that these two sympatric *Borrelia* species rarely co-occur in the same nymphal tick^54^,^55^,^56^. The host-specificity of *B. afzelii* for rodents and *B. garinii* for birds is believed to be mediated by the complement system of the vertebrate host^15^,^16^,^55^,^56^. *In vitro* assays have shown that *B. afzelii* is tolerant to rodent complement but is lysed by bird complement, and vice versa for bird-adapted *Borrelia* species like *B. garinii* and *B. valaisiana*^15^,^16^. However, as mentioned previously, there are very few *in vivo* studies showing that *B. afzelii* spirochetes are killed in nymphs that feed on avian hosts^13^. Two recent studies that quantified the abundance of rodent- and bird-adapted *Borrelia* species in wild questing *I. ricinus* nymphs provided indirect evidence for the complement hypothesis^54^,^57^. In the first study, the spirochete load of nymphs co-infected with rodent- and bird-adapted *Borrelia* species was significantly lower than the additive expectation of when the species occurred alone^54^. In the second study, co-infections between *B. afzelii* and *B. garinii* were surprisingly common in wild nymphs, however, the spirochete load of the dominant *Borrelia* species was always an order of magnitude higher than the sub-dominant species^57^. Taken together, these two studies provide indirect evidence that some component of the vertebrate blood meal (e.g. complement) was reducing the spirochete load of the mal-adapted *Borrelia* species^54^,^57^. Thus co-infections between rodent- and bird-adapted *Borrelia* species in *I. ricinus* nymphs may be much more common than previously thought but the spirochete population of one of the two species is probably dead.

Migratory songbirds have a great capacity to disperse ticks and tick-borne pathogens to new geographic locations^58^. Interestingly, phylogenetic studies have shown that *B. afzelii* has much more spatial genetic structure than *B. garinii*, which may reflect the migratory potential of their rodent and bird hosts^59^,^60^. Our literature review found that ground-dwelling birds such as the blackbird, song thrush, European robin and dunnock were common carriers of *B. afzelii*-infected immature *I. ricinus* ticks. These studies have led to speculation that *B. afzelii* can use bird hosts to achieve transmission and is not as restricted to rodent hosts as previously thought^61^. However, all of these studies used PCR-based methods to determine *Borrelia* infection and none of these studies used culture-based methods to show that the spirochetes are actually alive. The present study shows that nymph-to-adult transtadial transmission of *B. afzelii* DNA can occur on birds but that the spirochetes are not necessarily viable. We suggest that PCR-based studies demonstrating that birds can amplify *B. afzelii* or that rodents can amplify *B. garinii* should be interpreted with great caution.

We propose three alternative explanations for the observation that *B. afzelii*-positive larval ticks are regularly collected from wild birds (Table 1). First, the larval ticks could have acquired *B. afzelii* via vertical transmission. There is a general consensus that vertical transmission in *Ixodes* ticks is rare for *B. burgdorferi* s. l. pathogens but common for the relapsing fever spirochete *B. miyamotoi*^25^,^26^. A second explanation is partial blood feeding where larval ticks take multiple meals from different vertebrate hosts. Host blood meal analysis of wild *I. ricinus* ticks in Switzerland suggests that 9.5–19.5% of larval ticks feed on multiple hosts^62^,^63^. An early study on *B. burgdorferi* s. s. in *I. scapularis* showed that partially fed larval ticks could acquire spirochetes^64^. Thus larval ticks could acquire *B. afzelii* from a partial blood meal on a rodent and then attach to a bird to feed to repletion. A recent study in the Netherlands reported that wild *I. ricinus* larvae carried *B. afzelii* (prevalence was 0.62%), and these larvae were able to infect pathogen-free rodents^27^. The authors suggested that their data were consistent with both vertical transmission and partial blood meals^27^. A third explanation involves variation in the efficiency of co-feeding transmission between strains of *B. afzelii*. Like many vector-borne pathogens, populations of *B. afzelii* consist of multiple strains^57^,^65^,^66^,^67^,^68^. Two recent studies found that some *B. afzelii* strains are much more efficient at co-feeding transmission than other strains^32^,^34^. The *B. afzelii* strains in the blackbird experiment were derived from naturally infected *Apodemus* mice, and their genetic identity and co-feeding transmission efficiency on rodent hosts are currently unknown. For this reason, we used *B. afzelii* isolate NE4049 in the great tit experiment because it has a high efficiency of co-feeding transmission (>50%) on lab mice^32^,^34^.

We conclude that blackbirds and great tits do not allow efficient co-feeding transmission of viable *B. afzelii* spirochetes. The present study supports the hypothesis that the bird complement system inhibits the rodent-adapted *B. afzelii* from exploiting avian hosts for spirochete transmission. The generality of our results for other combinations of *B. afzelii* strains and bird species remains to be investigated.

## Methods

### Birds

Eurasian blackbirds and great tits are two abundant bird species in Europe. The Eurasian blackbird is frequently infested with tens of immature *I. ricinus* ticks^24^,^69^,^70^. The great tits in our Belgian study population frequently carry high burdens of immature *I. ricinus* ticks (maximum number of larvae = 40; nymphs = 17)^71^,^72^. Both bird species are competent reservoir hosts for bird-adapted *B. burgdorferi* s. l. pathogens. Blackbirds transmit *B. garinii, B. valaisiana*, and *B. turdi*^24^,^40^,^41^, whereas great tits transmit *B. garinii*^12^,^73^,^74^,^75^.

Four pathogen-free blackbirds and four pathogen-free great tits were obtained, respectively, from a certified Belgian breeder and a laboratory colony at the Netherlands Institute of Ecology (NIOO-KNAW)^76^. Environmental conditions consisted of a 12 h light: 12 h dark cycle (7:00 to 19:00) and ambient temperature varied with outdoor conditions. Birds were given food and water *ad libitum*, and had access to a fresh water bath. Birds were kept in individual cages and were allowed to habituate to the lab environment for at least four days before the start of the experiment.

#### ***Ixodes ricinus*** ticks

Pathogen-free *I. ricinus* larval ticks from the laboratory colony at the University of Neuchâtel were fed on *B. afzelii*-infected rodents and were allowed to moult into *B. afzelii*-infected nymphs (hereafter referred to as the challenge nymphs). The creation of the challenge nymphs was different for the blackbirds and great tits (see below). The pathogen-free *I. ricinus* larvae that were used for co-feeding with the infected challenge nymphs were obtained from a German laboratory colony (IS Insect Services GmbH, Berlin).

For the blackbirds, the challenge nymphs had been fed as larval ticks on 7 field-captured and naturally infected wood mice (*Apodemus sylvaticus* L.). Infection with *B. burgdorferi* s. l. of each wood mouse was confirmed with a commercial Lyme borreliosis ELISA assay and qPCR on an ear tissue sample, using protocols described elsewhere^77^. All challenge nymphs were kept in individual Eppendorf tubes to facilitate random sampling. We randomly selected 9–10 nymphs from each of the 7 *Apodemus* mice and tested them for *B. afzelii* infection using qPCR. The infection prevalence of the challenge nymphs used in the black bird experiment was 68.1% (47 infected/69 total).

For the great tits, the challenge nymphs had been fed as larval ticks on 15 *Mus musculus* BALB/c mice that had been experimentally co-infected via tick bite with *B. afzelii* isolates NE4049 and Fin-Jyv-A3. Infection with *B. afzelii* of each mouse was confirmed with a commercial Lyme borreliosis ELISA assay and qPCR on an ear tissue sample, using protocols described elsewhere^77^. Isolates Fin-Jyv-A3 and NE4049 were obtained from a bank vole (*Myodes glareolus*) in Finland and an *I. ricinus* nymph in Switzerland. Isolate Fin-Jyv-A3 carries *ospC* major group (oMG) A3. Isolate NE4049 has multi-locus sequence type 679, oMG A10, and strain ID number 1887 in the *Borrelia* MLST database^11^,^32^,^34^,^77^. We used isolate NE4049 (also referred to as *ospC* strain A10) because it has very efficient co-feeding transmission in lab mice^11^,^32^,^34^. All challenge nymphs were kept in individual Eppendorf tubes to facilitate random sampling. We randomly selected 7–10 nymphs from each of the 15 mice and tested them for *B. afzelii* infection using a previously described qPCR protocol^77^. The infection prevalence of the challenge nymphs used in the great tit experiment was 91.5% (130 infected/142 total), of which 75.4% (107/142) and 59.9% (85/142) were infected with isolates NE4049 and Fin-Jyv-A3, respectively.

### Ethics statement and animal experimentation permits

Experiments on the birds were carried out at the University of Antwerp, Belgium in accordance with national environmental legislation and university regulations. The Ethics Committee for Animal Experiments of the University of Antwerp approved the tick infestation procedure (Dossier 2009-32) and the transmission experiment (Dossier 2014-49). Experiments to create the *I. ricinus* nymphs infected with *B. afzelii* were carried out at the University of Neuchâtel, Switzerland. The commission that is part of the ‘Service de la Consommation et des Affaires Vétérinaires (SCAV)’ of Canton Vaud, Switzerland evaluated and approved the ethics of this part of the study. The Veterinary Service of the Canton of Neuchâtel, Switzerland issued the animal experimentation permits (NE1/2014 and NE4/2016).

### Study design

**Figure 1:**
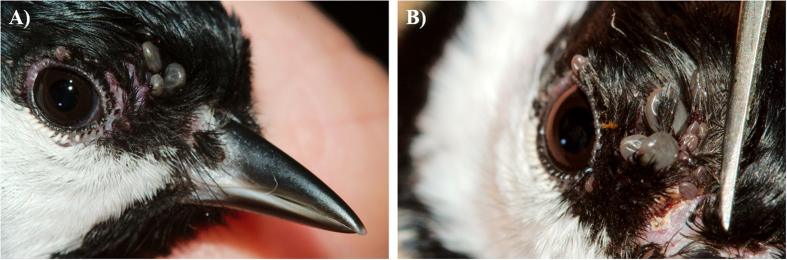
Naïve *I. ricinus* larvae co-feed with *B. afzelii*-infected nymphs on the head of a great tit. The larvae (small) and nymphs (large) were placed underneath the crown-feathers on the right side of the head (**A**: lateral; **B**: frontal view). By feeding in close spatial and temporal proximity, the *B. afzelii* spirochetes can migrate directly from the infected nymphs to the naïve larvae via co-feeding transmission. Dr. Frank Adriaensen took the photos.

The infestation experiments for the blackbirds and great tits were conducted in November 2015 and February 2016, respectively. For each bird species, four individuals were infested with 11–12 *B. afzelii*-infected *I. ricinus* nymphs that had been randomly selected from the pool of available nymphs. These tick loads are within the range observed in field-captured birds^24^,^69^,^70^,^71^,^72^. Nymphs were placed underneath the crown feathers on the right side of the head above the eye using moistened tweezers, as described in ref. 72 (Fig. 1). After each infestation, birds were kept for 1 h in an air-permeable cotton bag (size: 25 cm × 20 cm for blackbirds; 20 cm × 15 cm for great tits) inside a darkened cage to keep them inactive and to facilitate tick attachment^72^. Twenty-four hours after nymphal exposure, the blackbirds and great tits were additionally infested with 40–50 xenodiagnostic larvae, following the same protocol as for the challenge nymphs. The larvae were placed near the nymphs to facilitate co-feeding transmission^32^,^33^,^34^. After each infestation, the cotton bags were checked for ticks to determine the number of nymphs and larvae that had attached to each bird. Birds were not checked for the number of attached nymphs to avoid disturbing these ticks. Following infestation, birds were returned to their individual cages (40 cm × 80 cm) that had a wire mesh floor to facilitate the daily collection of engorged ticks. Most of the engorged ticks were placed in 80% ethanol and stored at −20 °C. The remaining engorged ticks were allowed to moult to the next stage to study transstadial transmission of *B. afzelii* DNA. These ticks were kept in individual tubes under summer conditions (16 h light at 25 °C, 8 h at dark at 16 °C) and with a relative humidity >90%. For the great tit experiment, we further tested whether the *B. afzelii* spirochetes in the adult ticks were actually viable. Each of five adult ticks that had fed as challenge nymph on three great tits, were cut into two halves using sterile scissors. One tick half was screened for *B. afzelii* infection using qPCR, the other tick half was cultured in tubes containing BSK-II medium^78^, incubated at 34 °C, and examined by dark-field microscopy every 10 days for 40 days.

### **Probability that each bird was challenged by at least one** ***B. afzelii*** **-infected nymph**

If avian blood clears spirochetes from feeding nymphs, the post-hoc analysis of such ticks is not a reliable indicator as to whether the bird was challenged or not. For example, after feeding *B. afzelii*-infected *I. ricinus* nymphs on pheasants, 0 of the 56 engorged nymphs tested positive for *B. afzelii*^13^. In this case, it is critical to know the prevalence of *B. afzelii* infection in the flat nymphs (q) before they are placed on the birds, and the number of nymphs that attached to the bird (n). With this information one can calculate the probability (P) that each bird was bitten by at least one *B. afzelii*-infected challenge nymph as follows: P = 1 − (1 − q)^n^. The exact value of n is often unknown: the maximum is the number of nymphs that attached to the bird (n_max_) and the minimum is the number of blood-engorged nymphs that were recovered (n_min_). For example, for a bird that was infested with 12 challenge nymphs with an expected prevalence of infection of 0.681 and for which 4 engorged challenge nymphs were recovered, the probability that at least one of the challenge nymphs was infected with *B. afzelii* ranges from P_max_ = 0.9999989 to P_min_ = 0.9896447.

### **PCR-based detection of** ***B. afzelii***

Total tick DNA was purified using the DNeasy Blood & Tissue Kit following the protocol for the purification of total DNA from ticks. All ticks were screened for the presence of *B. burgdorferi* s. l. using a duplex qPCR that was designed based on existing qPCR protocols that target fragments of the *ospA* gene^79^ and the *flagellin* gene^80^. A detailed description of primers, probes and the qPCR protocol is given in an earlier study^75^. For the subsample of qPCR-positive ticks that had fed on the blackbirds, the *B. burgdorferi* s. l. genospecies was determined by PCR amplification and sequencing of the *ospC* gene^81^ and the variable 5S-23S (rrfA-rrlB) intergenic spacer (IGS) region gene^75^. For each PCR and multiplex qPCR, positive controls, negative controls, and blank samples were included. To minimize contamination, the three steps of the PCR protocol were performed in separate rooms. The DNA extraction room was kept at negative pressure, whereas the reagent setup and sample addition rooms were kept at positive pressure. All rooms had airlocks.

### Literature review

We used an extensive systematic literature search that is described in Hofmeester *et al*. (2016)^82^. The search strings and selection procedure as well as the dataset are provided in the supplementary material of that study (URL: http://iopscience.iop.org/article/10.1088/1748-9326/11/4/043001/meta). The search was done using PubMed, Web of Science and Scopus to review the occurrence of *B. burgdorferi* s. l. pathogens in Europe, in songbird hosts and their *I. ricinus* ticks. The last literature search was carried out in January 2015 and used the years 1945–2014. We added one more study to that dataset^22^. Only studies that identified the *Borrelia* genospecies in infected larvae and nymphs derived from songbirds were included, which resulted in 19 usable studies.

## Additional Information

**How to cite this article**: Heylen, D. J. A. *et al*. Inefficient co-feeding transmission of *Borrelia afzelii* in two common European songbirds. *Sci. Rep.* **7**, 39596; doi: 10.1038/srep39596 (2017).

**Publisher's note:** Springer Nature remains neutral with regard to jurisdictional claims in published maps and institutional affiliations.

## Supplementary Material

Supplementary Information

## Figures and Tables

**Table 1:** *Borrelia afzelii* infections have been found in *Ixodes ricinus* larvae and nymphs feeding on many different species of birds.

Bird Species	*Ixodes ricinus* larvae				*Ixodes ricinus* nymphs			
	# studies reporting *B. afzelii* infections	# birds tested	# ticks tested	# infected ticks	# studies reporting *B. afzelii* infections	# birds tested	# ticks tested	# infected ticks
*Anthus trivialis*					1 (53)	120	85	4
*Carduelis cabaret*					1 (22)	**	5	1
*Carduelis chloris*					1 (19)	1	3	1
*Coccothraustes coccothraustes*					1 (53)	2	2	1
*Erithacus rubecula*	2 (52,61)	124	38*	8	5 (19,22,61,73,83)	316	366	11
*Fringilla coelebs*	1 (53)	37	42	1	2 (19,53)	52	50	6
*Locustella naevia*					1 (73)	2	5	1
*Motacilla cinerea*	1 (73)	3	1	1	1 (73)	3	9	2
*Parus major*	2 (73,75)	187	266	3	4 (19,20,73,75)	220	403	15
*Phoenicurus phoenicurus*					1 (22)	**	38	1
*Phylloscopus trochilus*					1 (22)	**	37	2
*Prunella modularis*					5 (19,22,24,73,83)	87	430	27
*Saxicola rubetra*					1 (22)	**	2	1
*Sylvia atricapilla*					1 (24)	16	18	1
*Sylvia communis*					2 (53,73)	12	13	4
*Sylvia curruca*					1 (22)	**	22	2
*Troglodytes troglodytes*	1 (83)	4	5	1				
*Turdus iliacus*	1 (61)	19	4	1	2 (53,61)	28	60	5
*Turdus merula*	1 (61)	11	2	1	7 (19,22,23,24,53,61,73)	141	1009	35
*Turdus philomelos*					6 (19,22,24,52,53,73)	131	436	11
*Turdus viscivorus*					1 (53)	2	2	1

Data are from a literature search that included 19 publications that report on *Borrelia* genospecies in bird-derived ticks.

^*^One study did not report on the total number of larvae that were screened, therefore this number is an under-estimation.

^**^Study did not report on the total number of captured birds.

**Table 2:** *Borrelia afzelii* infection status is shown for the *Ixodes ricinus* ticks that had co-fed on two species of songbird, the blackbird (*Turdus merula*) and the great tit (*Parus major*).

Species	Bird N°	Nymphs			Larvae	
		Engorged	Moulted	Attached**	Engorged	Moulted
		infect./total	infect./total		infect./total	infect./total
*T. merula*	1 - ♂	N.A.	0/2	9	0/10	0/7
*T. merula*	2 - ♀	N.A.	4/5	12	0/10	0/15
*T. merula*	3 - ♀	N.A.	0/4	11	0/9	0/6
*T. merula*	4 - ♂	N.A.	2/6	12	0/10	0/23
*P. major*	1 - ♀	2/2	0/1*	4	0/14	N.A.
*P. major*	2 - ♀	2/2	1/1*	8	0/24	0/9
*P. major*	3 - ♂	5/5	3/3*	10	2/22	0/2
*P. major*	4 - ♂	2/2	N.A.	6	1/16	0/3

The blood-engorged nymphs and larvae were either placed in ethanol following drop-off or allowed to moult into the next stage (adult and nymph, respectively). All engorged and moulted ticks were screened for *B. afzelii* infection using qPCR. Adult ticks were also cultured in BSKII-medium to test for nymph-to-adult transtadial transmission of viable *B. afzelii* spirochetes.

^*^Engorged nymphs were allowed to moult into adult ticks and were cut in half. One half was screened for *B. afzelii* using qPCR and the other half was cultured in BSK II-medium to test for viable spirochetes. None of them yielded spirochete cultures; therefore *B. afzelii* is not capable of transstadial transmission in the presence of bird blood.

^**^Attached = total number of nymphs placed on the bird minus the number of nymphs left in the bag.
